# Functional analysis of the *GRMZM2G174449* promoter to identify *Rhizoctonia solani*-inducible *cis*-elements in maize

**DOI:** 10.1186/s12870-017-1181-5

**Published:** 2017-12-04

**Authors:** Fangfang Yang, Xinhua Ding, Jing Chen, Yanting Shen, Lingguang Kong, Ning Li, Zhaohui Chu

**Affiliations:** 1 State Key Laboratory of Crop Biology, College of Agronomy, Shandong Agricultural University, Tai an, 271018 Shandong Province People’s Republic of China; 2 Shandong Provincial Key Laboratory of Agricultural Microbiology, College of Plant Protection, Shandong Agricultural University, Tai an, 271018 Shandong Province People’s Republic of China

**Keywords:** Maize, Banded leaf and sheath blight, *Rhizoctonia solani*, *cis*-element, Genetic engineering

## Abstract

**Background:**

Banded leaf and sheath blight (BLSB), caused by the necrotrophic fungus *Rhizoctonia solani*, is a highly devastating disease in most maize and rice growing areas of the world. However, the molecular mechanisms of perceiving pathogen signals are poorly understood in hosts.

**Results:**

Here, we identified a *Rhizoctonia solani*-inducible promoter *pGRMZM2G174449* in maize. Deletion analysis showed that the −574 to −455 fragment was necessary for *pGRMZM2G174449* in responding to *R. solani* and this fragment contained the unknown pathogen-inducible *cis-*elements according to the bioinformatics analysis. Furthermore, detailed quantitative assays showed that two *cis*-elements, GCTGA in the −567 to −563 region and TATAT in the −485 to −481 region, were specifically responsible for the *R. solani*-inducible activity. A series of point mutation analysis indicated that the two *cis*-elements have the conserved motifs of NHWGN and DWYWT, respectively.

**Conclusion:**

Our results indicated that *pGRMZM2G174449* is a good *R. solani*-inducible promoter suitable for genetic engineering of BLSB resistance. And NHWGN and DWYWT are two *R. solani*-inducible *cis*-elements that play important roles in *pGRMZM2G174449* responding to *R. solani*.

**Electronic supplementary material:**

The online version of this article (10.1186/s12870-017-1181-5) contains supplementary material, which is available to authorized users.

## Background

Pathogen attacks have drastic effects on crop growth and development, which significantly limit agricultural productivity. To enhance crop disease resistance, cloning of key genes related to pathogen invasion and precisely regulating transgene expression are important measures for solving the problem [1]. In current applications, constitutive promoters, such as the cauliflower mosaic virus 35S (CaMV35S) and ubiquitin promoters, have been frequently used to assess the effects of transgene expression in many plant species. However, in certain cases, the constitutive overexpression of defense-related genes may result in negative effects on crop growth and yields [2–4]. Therefore, cloning and identifying characterizing pathogen-inducible promoters are the keys to understanding the regulation mechanisms of defense-related genes, and these promoters could be the most useful types of promoters for engineering crop lines with enhanced and durable disease resistance [5].

Many pathogen-inducible promoters have been identified [6–8], and some of them have been used to generate high-quality transgenic plants [9–11]. Pathogen-inducible promoters usually possess many conserved *cis*-elements that are potential binding sites for pathogen-responsive transcription factors. Among them, the GCC-like elements [12, 13] and the W-box [14–16] are two groups of *cis*-regulatory elements that have been widely studied and functionally validated. Beside the GCC-like elements and W-box, some other pathogen-inducible *cis*-elements have also been identified, such as the S-box [17], G-box [18], E-box [19], PRE2 and PRE4 [20] as well as the MYB recognition elements [8]. Some of these elements, such as the W-box, GCC-box and S-box, have been used to construct synthetic pathogen-inducible promoters [9, 21].

Banded leaf and sheath blight (BLSB), which is caused by the necrotrophic fungal pathogen *Rhizoctonia solani*, often leads to extensive necrosis in the leaf sheaths of hosts, eventually causing the death of the infected plant and resulting in substantial economic losses. It is a typical soil-born and annual cumulative disease which widely spread out in most maize and rice growing areas of the world [22, 23]. To date, sheath blight resistance has been demonstrated to be controlled by minor-effect QTLs [24, 25], and only a few defense-related genes have been identified [11, 26–29]. Recent study showed that OsASR2 could regulate the response of *Os2H16* gene to *R. solani* by targeting the GT-1 *cis*-element [30]. However, understanding of the regulation mechanisms of *R. solani*-inducible genes is still very limited, with a few *R. solani*-inducible promoters and three *cis*-elements have been reported recently [30–34].

In this study, we focused on a maize *R. solani*-induced expression gene, *GRMZM2G174449*. To better understand how the *GRMZM2G174449* gene is regulated, we completely analyzed the promoter of *GRMZM2G174449* by using β-glucuronidase (*GUS*) or green fluorescent protein (*GFP*) gene as reporter genes. We found that the 5′-flanking sequence of the *GRMZM2G174449* promoter could be induced by *R. solani* inoculation. Deletion analysis showed that two novel *cis*-elements, GCTGA and TATAT, were specifically responsive to *R. solani* infection. Mutation analysis indicated that these two elements have the conserved motifs of NHWGN and DWYWT, respectively. These results will improve our understanding of *GRMZM2G174449* regulation and increase the number of promoter and *cis*-elements available for potential use in development of transgenic plants with enhanced *R. solani* resistance.

## Methods

### Plant materials and pathogens

Rice cultivar Zhonghua 11 and maize inbred line B73 were grown in greenhouse at 28 °C with a 16/8 h light/dark cycle. *Nicotiana benthamiana* (NB) was grown in chamber at 25 °C under a 16/8 h light/dark cycle. For the tissue expression analysis of *GRMZM2G174449* and its promoter assay, tissues were harvested for total RNA isolation and GUS assay, including with young root, young culm, young leaf, root, culm, leaf, anther, pistil and endosperm tissues. As previously reported [34], *R. solani* strains were grown in Potato-Dextrose-Broth media (potato at 200 g/L, glucose at 20 g/L and agar at 15 g/L) at 25 °C for 3 days. *Magnaporthe grisea* strain RB21 was grown in Rice Bran media (rice bran at 20 g/L, yeast powder at 2 g/L and agar at 15 g/L) at 25 °C for 10 days. *Xanthomonas oryzae* pv. *oryzae* strain PXO99 and *Xanthomonas oryzae* pv. *oryzicola* strain RS105 were grown in Polypeptone-Sucrose-Agar media (polypeptone at 10 g/L, glutamic acid at 1 g/L, sucrose at 10 g/L and agar at 15 g/L) at 28 °C for 2 days and then suspended in sterile water to OD_600_ = 0.5. Infected and non-infected leaves were harvested for the GUS and GFP assays.

### Vector constructions of the *GRMZM2G174449* promoter and its deletion derivatives

The full-length *GRMZM2G174449* promoter was amplified from the maize inbred line B73 based on the maize genome sequence (http://www.maizegdb.org/gbrowse/maize_v4) with the primers listed in Additional file 1: Table S1. To generate the *GRMZM2G174449* promoter assay construct, the appropriate restriction sites were introduced into the PCR-amplified promoter (*Sal* I at the 5ˊ end; *Bam*H I at the 3ˊ end), and then cloned into double digested *pCAMBIA1391-Sal* I-*Bam*H I-cut, which was named pC1391 D0. The deleted promoters were cloned into the *pCXGUS-P* and *pCXGFP-P* vectors as previously described [30, 35] to generate deletion constructs containing various fragments (−1518 to +46, pCXGUS D1; −1114 to +46, pCXGUS D2; −694 to +46, pCXGUS D3; −454 to +46, pCXGUS D4; −274 to +46, pCXGUS D5; −574 to +46, pCXGUS D6; −574 to −455, pCXGFP delA; −574 to −550, pCXGFP delB; −549 to −528, pCXGFP delC; −527 to −491, pCXGFP delD; −490 to −478, pCXGFP delE; −477 to −455, pCXGFP delF). To identify the GCTGA and TATAT *cis*-elements, the sequence of two elements were repeated twice, fused with the 35S minimum promoter and named 2 × GCTGA and 2 × TATAT, respectively. For mutation analysis, the mutations of GCTGA and TATAT were repeated twice and fused with the 35S minimum promoter as described above. All the primers were listed in Additional file 1: Table S1.

### Rice transformation

For promoter analysis, the pC1391 D0, 2 × GCTGA and 2 × TATAT constructs were transformed into rice cultivar Zhonghua 11 to generate the transgenic plants. *Agrobacterium tumefaciens*-mediated method was used for rice transformation with mature embryos and *A. tumefaciens* strain EHA105 [36]. Positive selection and validation were performed with PCR basing on *GUS* or *GFP* genes, and three T_1_ lines of each were used for further analysis.

### Transient expression in *Nicotiana benthamiana* and quantification of GUS and GFP

Transient expression in NB leaves was performed according to a previously described method [30, 34]. Histochemical GUS staining of transgenic rice leaves was performed as described previously [37]. The leaves were immersed in staining buffer, that is 0.1 M sodium phosphate buffer (pH = 7.0) containing 1 mg/ml X-Gluc, 0.5 mM K_3_[Fe(CN)_6_], 0.5 mM K_4_[Fe(CN)_6_], 10 mM Na_2_EDTA, 0.1% (*v*/v) Triton X-100, and 10% (v/v) methanol, for 24 h at 37 °C in the dark. Quantitative fluorometric GUS assays were performed by incubating the extracts with the 4-methyl-umbelliferyl-β-D-glucuronide (MUG) substrate in a lysis buffer for 15 min at 37 °C. GFP fluorescence was observed under a Leica M205C stereo microscope (Leica, Germany), and fluorescence was quantified using an EnSpire Multimode Plate Reader (PerkinElmer, USA) as described in a previous study [30, 38]. The GFP fluorescence was excited at wavelength of 480 nm and measured at 520 nm.

### RNA isolation and expression pattern analysis

Total RNA was isolated using Plant RNA Kit according to the manufacturer’s instructions (OMEGA Bio-tek, USA). For cDNA synthesis, we used the SuperQuickRT MasterMix Kit (CWBIO, Beijing, China) with 2 μg of total RNA as a template in a 20-μL reaction mixture. Quantitative real-time PCR (qRT-PCR) was performed with an UltraSYBR Mixture Kit (CWBIO, Beijing, China) using the QuantStudio™ 6 Flex Real-Time PCR System (Life Technologies, USA). The relative expression level of each gene was calculated with normalizing to *Actin1* (Accession NO. GQ339773) mRNA levels. Changes in expression were calculated using the ΔΔ Ct method. The gene-specific primers used are listed in Additional file 1: Table S1.

### Statistical analysis

All data analyses were repeated three times with three replicate experiments independently. Standard deviations were indicated by error bars and the statistical significances were determined by one-way variance analysis. The mean differences were compared using Student’s t test.

## Results

### Characterization of *R. solani*-inducible gene *GRMZM2G174449*

**Fig. 1:**
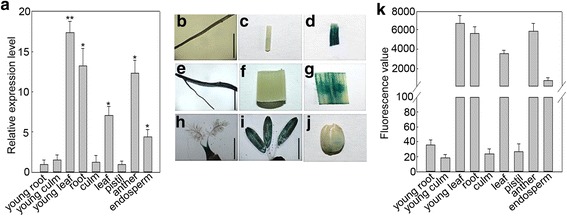
Tissue expression analysis of the maize gene *GRMZM2G174449* and its promoter. **a** Tissue-specific analysis of *GRMZM2G174449* in maize. The significant differences were compared with young root. Asterisks indicate statistically significant differences, as determined by Student’s t-tests (*P < 0.05, **P < 0.01). **b-j** GUS histochemical staining in different tissues of transgenic rice. **b** young root; **c** young culm; **d** young leaf; **e** root; **f** culm; **g** leaf; **h** pistil; **i** stamen; **j** endosperm. Bars = 5 mm. **k** Quantitative GUS assays of different tissues in transgenic rice

**Fig. 2:**
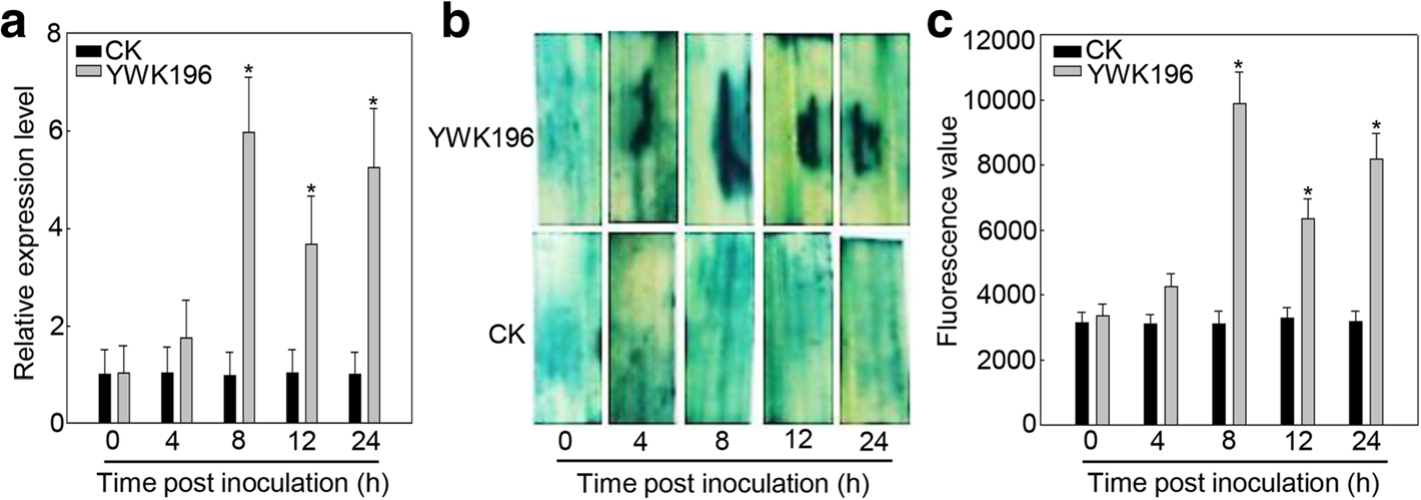
Pathogen-inducible expression pattern of the maize gene *GRMZM2G174449* and its promoter. **a** Time course of *GRMZM2G174449* expression after *R. solani* infection in 21-day-old seedlings. Asterisks indicate statistically significant differences, as determined by Student’s t-tests (*P < 0.05). **b** GUS histochemical staining in transgenic rice plants post inoculation with *R. solani* strain YWK196. **c** Quantitative GUS assays in transgenic rice leaves plants post inoculation with *R. solani* strain YWK196. Asterisks indicate statistically significant differences, as determined by Student’s t-tests (*P < 0.05)

In our RNA-Seq data (Accession NO. SRP076058), *GRMZM2G174449* was one of the up-regulated genes induced by *R. solani* strain YWK196 in B73. The expression patterns of tissue specific were further analyzed by qRT-PCR. As shown in Fig. 1a, *GRMZM2G174449* was expressed at higher levels in young leaf, root, leaf, anther and endosperm tissues, but the gene exhibited low levels in other tissues of maize B73. A 6-fold increase of *GRMZM2G174449* expression was observed 8 h post inoculation (hpi) with *R. solani* strain YWK196, with the expression levels maintained at 24 hpi (Fig. 2a), suggesting that the *GRMZM2G174449* promoter could respond to *R. solani* in B73.

### Bioinformatic analysis of the *GRMZM2G174449* promoter

To identify the regulatory mechanisms of the *GRMZM2G174449* gene, we isolated its promoter region (−1954 to +46, Accession NO. KY086278). According to the PLACE database [39], some putative *cis*-elements are predicted in *pGRMZM2G174449* (Additional file 2: Figure S1). The TATA-box (5ˊ-TATAAA-3ˊ) starts 76 bp upstream of the ATG and 30 bp upstream of the transcription start point (TSP), while a CAAT-box was located close to the TATA-box, which was 53 bp upstream of the TSP. In addition, the *GRMZM2G174449* promoter also contains one pathogen- and salt-inducible element GT-1, GAAAAA [30, 40], five methyl jasmonate (MeJA)-responsive elements, CGTCA [41], two gibberellic acid (GA)-responsive elements, TAACAA [42], three abscisic acid (ABA)-responsive elements, ACGTG [43] and one auxin-responsive element, TGTCTC [44]. Eleven CANNTG elements which are known to be the binding site of the basic helix-loop-helix (bHLH) transcription factor [45], and one W-box (TTGACC) which is specifically recognized by the WRKY DNA binding proteins [46], were found in the promoter region.

### The *GRMZM2G174449* promoter derived expression pattern

By using *A. tumefaciens*-mediated transformation, transgenic rice plants carrying the *GRMZM2G174449* promoter-*GUS* construct were obtained. First, we examined the tissue-specific expression pattern of the *GRMZM2G174449* promoter in transgenic rice. As shown in Fig. 1b-k, the GUS staining showed stronger enzymatic activity in the young leaf, root, leaf, anther and endosperm tissues than other tissues in the transgenic plants. Consistent with the GUS staining results, the *GUS* gene was expressed at high level in the young leaf, root, leaf, anther and endosperm tissues. Also, the rice result was similar to the tissue-specific expression pattern of *GRMZM2G174449* gene in maize B73 (Fig. 1a).

We then examined the pathogen-inducible activity of the *GRMZM2G174449* promoter by *R. solani* in transgenic rice leaves. After treatment with *R. solani* strain YWK196, the GUS activity was enhanced approximately 3- fold by 8 hpi. It slowly declined by 24 hpi but remained higher than the control (Fig. 2b-c). This induction pattern was similar to the expression pattern in maize too (Fig. 2a).

### Deletion analysis of the *GRMZM2G174449* promoter

**Fig. 3:**
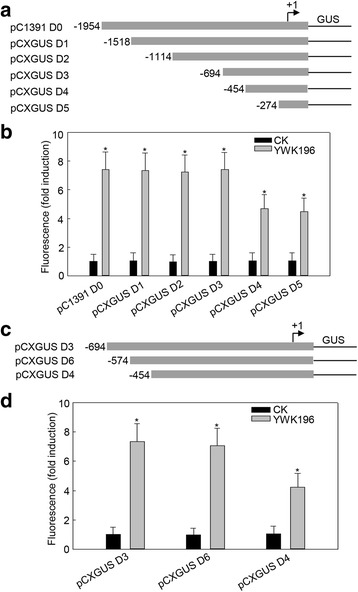
Quantitative fluorometric assays for GUS activity driven by various *GRMZM2G174449* promoter deletion constructs. **a** Diagram of various deletion derivatives of the *GRMZM2G174449* promoter. All promoter derivatives were fused to a *GUS* reporter gene. **b** The GUS activity of the DNA constructs in a NB transient expression system. The NB leaves were inoculated with *R. solani* strain YWK196 for 24 h. GUS activity was calculated relative to CK of the pC1391 D0 construct. Asterisks indicate statistically significant differences, as determined by Student’s t-tests (*P < 0.05). **c** Diagram of the deletion constructs in the −694 to −455 region of the *GRMZM2G174449* promoter. All promoter derivatives were fused to a *GUS* reporter gene. **d** The GUS activity of DNA constructs prepared in a NB leaf transient expression system. The NB leaves were inoculated with *R. solani* strain YWK196 for 24 h. GUS activity was calculated relative to CK of the pCXGUS D3 construct. Asterisks indicate statistically significant differences, as determined by Student’s t-tests (*P < 0.05)

To mine the specific regions of the *GRMZM2G174449* promoter that are responsive to *R. solani* treatment, a series of 5ˊ deletions were made in the *GRMZM2G174449* promoter region (Fig. 3a). Each construct was transiently expressed and induced by YWK196 inoculation in NB leaves, and GUS activity was assayed at 24 hpi. As shown in Fig. 3b, the full-length promoter (pC1391 D0) exhibited the highest level of inducible GUS activity, and equal induction levels were detected in constructs containing deletions up to −1518 (pCXGUS D1), −1114 (pCXGUS D2) and −694 (pCXGUS D3). However, this induction weakened in the construct containing deletion up to −454 (pCXGUS D4) and remained invariable in the pCXGUS D5 construct, but the induction levels in these two constructs were still higher than that in the control. These results indicated that the −694 to −455 and −274 to +46 fragments are two regions involved in the response to *R. solani*. Bioinformatic analysis of the two regions showed that the −694 to −455 region do not contain any known pathogen-inducible *cis*-elements, while the −274 to +46 region contains a *cis*-element GT-1 (GAAAAA) knowns as *R. solani*-inducible [30].

To identify the novel *R. solani*-inducible *cis*-elements in the −694 to −455 region, a 5ˊ deletion was further constructed in this region (Fig. 3c). These constructs were then tested in transient expression assays in NB leaves inoculated with YWK196 at 24 hpi. The construct with a deletion up to −574 (pCXGUS D6) showed almost equal GUS induction to that of the pCXGUS D3 construct. The pCXGUS D4 construct showed a roughly one-third reduction in GUS activity compared with that of the pCXGUS D3 and pCXGUS D6 constructs (Fig. 3d). These results indicated that the novel *R. solani*-inducible *cis*-elements are narrowly localized in the −574 to −455 region.

### Two independent DNA fragments in the −574 to −455 region are responsive to *R. solani*

**Fig. 4:**
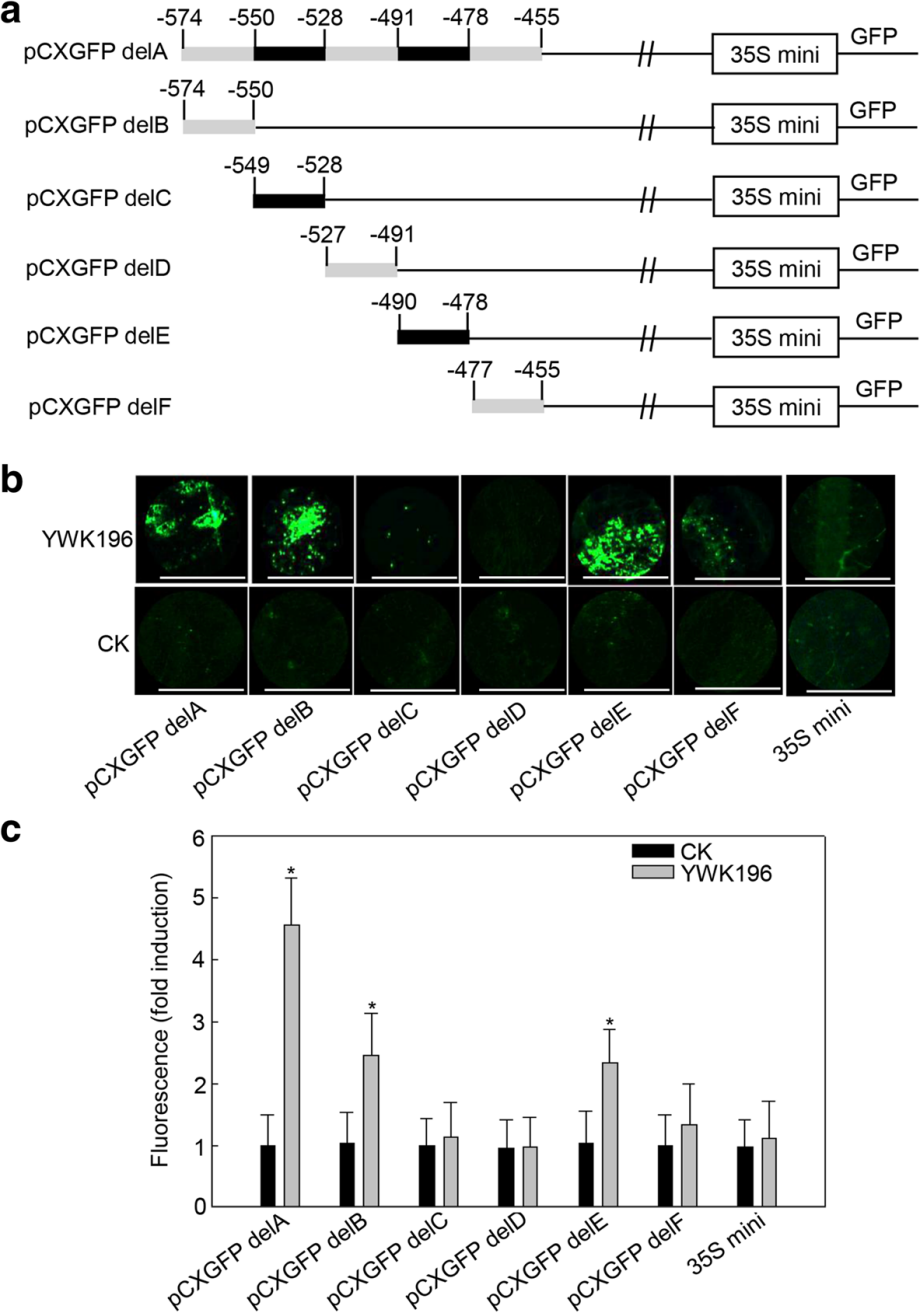
The −574 to −550 and −490 to −478 fragments in the −574 to −455 region are responsive to *R. solani*. **a** Schematic diagram of the −574 to −455 region (pCXGFP delA) and the five deleted derivatives (pCXGFP delB to pCXGFP delF) used to express *GFP* in tobacco leaves. **b** GFP fluorescence assay of young and expanded symmetrical NB leaves infiltrated with pCXGFP delA or its derivatives after *R. solani* strain YWK196 infection for 24 h. Bars = 5 mm. **c** Quantitative fluorometric assay of NB leaves. The fluorescence value was calculated relative to CK of pCXGFP delA. The 35S minimum promoter was used as the negative control. Asterisks indicate statistically significant differences, as determined by Student’s t-tests (*P < 0.05)

To narrow down the *R. solani*-inducible region of the −574 to −455 fragment, this region was further divided into five fragments of 10 to 30 bp in length, which were individually fused with the 35S minimum promoter (Fig. 4a). These constructs were then investigated in transient expression assays in NB leaves inoculated with YWK196 at 24 hpi (Fig. 4b-c). The pCXGFP delA construct showed GFP induction approximately 4.5-fold after treatment with YWK196, while the pCXGFP delB and pCXGFP delE constructs showed approximately 2.5-fold. In contrast, the pCXGFP delC, pCXGFP delD and pCXGFP delF constructs exhibited a very faint fluorescence signal. These results suggested that the 25-bp (GTACCTTGCTGATGGGCTCGGGGTG) and 13-bp (ACTACTATATCAA) sequences in the −574 to −455 region are responsive to *R. solani*.

### Characterization of the GCTGA and the TATAT are two *R. solani*-inducible *cis*-elements

**Fig. 5:**
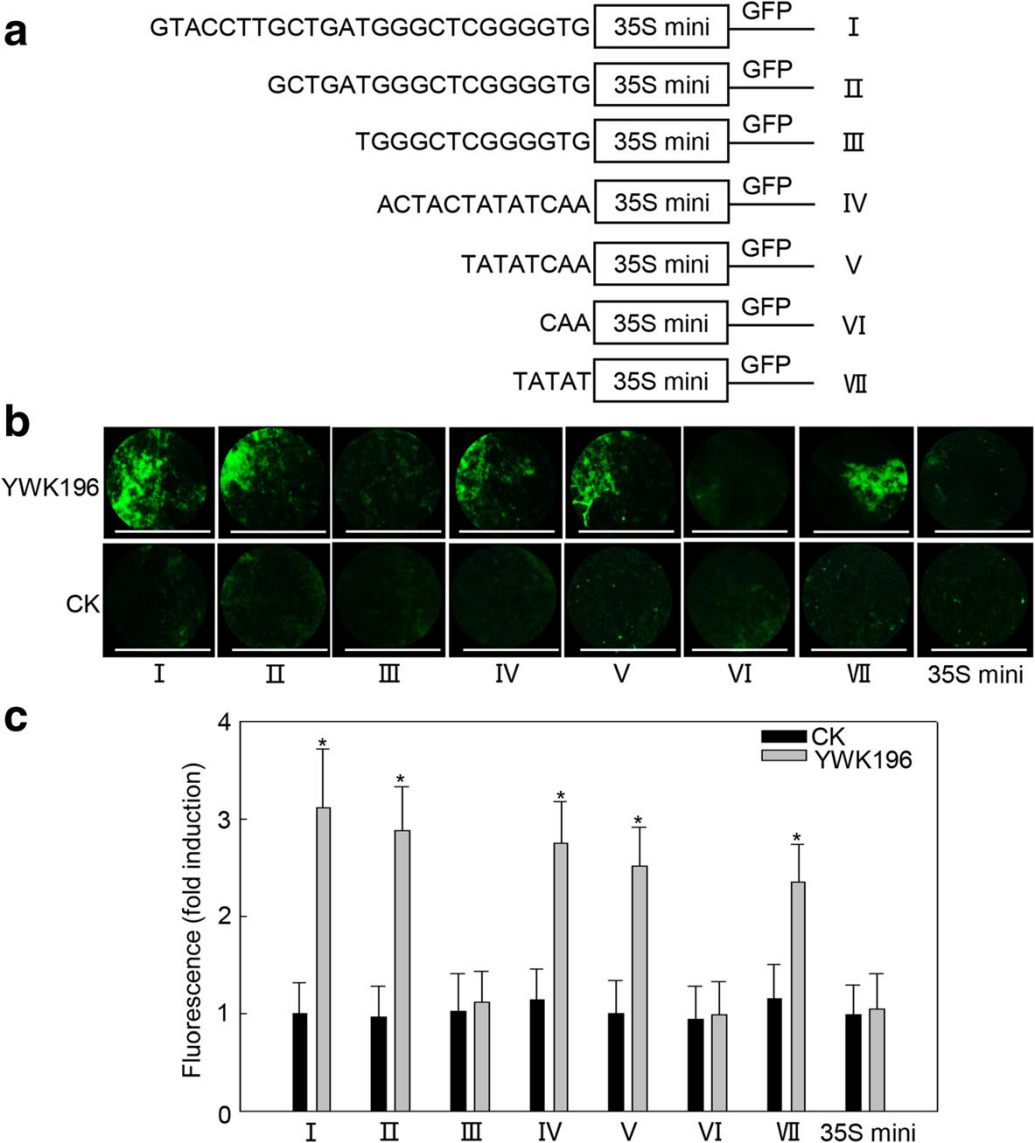
The GCTGA and TATAT are two *R. solani*-inducible *cis*-elements. **a** Schematic diagram of the −574 to −550 (I), −490 to −478 (IV) and corresponding deleted derivatives (II, III, V, VI, VII) used to express *GFP* in NB leaves. **b** GFP fluorescence assay of young and expanded symmetrical NB leaves infiltrated with the six constructs after *R. solani* strain YWK196 infection for 24 h. Bars = 5 mm. **c** Quantitative fluorometric assay of NB leaves. Fluorescence value was calculated relative to CK of I. The 35S minimum promoter was used as the negative control. Asterisks indicate statistically significant differences, as determined by Student’s t-tests (*P < 0.05)

In our parallel study, two novel *R. solani*-inducible *cis*-elements GTTGA and TATTT were identified in the other maize gene promoter, the *GRMZM2G315431* promoter [34]. Interestingly, homologous sequences of GCTGA and TATAT were found in the 25-bp (GTACCTTGCTGATGGGCTCGGGGTG) and the 13-bp (ACTACTATATCAA) DNA fragments, respectively. To identify whether the two sequences are core elements that involved in the response to *R. solani*, we produced other deletion derivatives fused with the 35S minimum promoter (Fig. 5a). The transient expression assays showed that deletion of GTACCTT in the 25-bp sequence exhibited an induction level (approximately 2.9-fold) nearly equal to that of the 25-bp sequence. However, the GFP induction was completely lost after deleting the GCTGA sequence (Fig. 5b-c). Examination on the 13-bp sequence showed that deletion of the ACTAC did not affect the GFP induction, with the remaining TATAT sequence maintaining an equal level after deletion of the CAA at the 3′ end. Also, deletion of TATAT resulted in no GFP induction (Fig. 5b-c). These results indicated that GCTGA and TATAT are necessary for the *R. solani* induction of the 25-bp and 13-bp sequences, respectively.

**Fig. 6:**
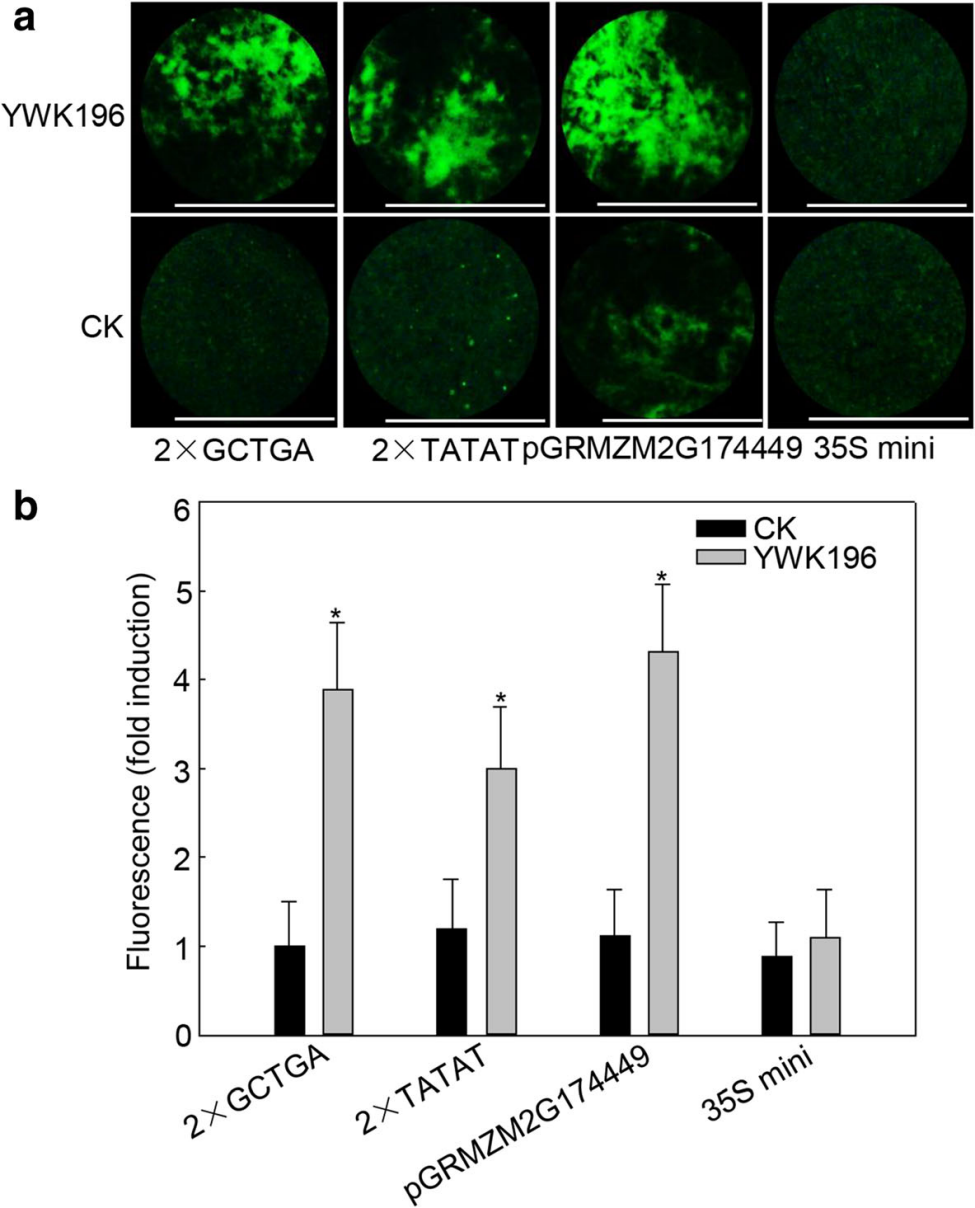
Determination of GCTGA and TATAT induction by *R. solani* in NB leaves. **a** GFP fluorescence assay of NB leaves expressing 2 × GCTGA and 2 × TATAT after treatment with *R. solani* strain YWK196 for 24 h. Bars = 5 mm. **b** Quantitative fluorometric assay of NB leaves. The fluorescence value was calculated relative to CK of 2 × GCTGA. The full-length *GRMZM2G174449* promoter and 35S minimum promoter were used as the positive and negative controls, respectively. Asterisks indicate statistically significant differences, as determined by Student’s t-tests (*P < 0.05)

To further determine whether these two *cis*-elements could respond to *R. solani* independently, we produced two constructs in which two tandem repeats of the two *cis*-elements (2 × GCTGA and 2 × TATAT) were fused to the 35S minimum promoter. The full-length *GRMZM2G174449* promoter and empty vector were used as controls. As shown in Fig. 6, the induction level of 2 × GCTGA (approximately 3.9-fold) was almost equal to that of the full-length *GRMZM2G174449* promoter (approximately 4.3-fold), and *R. solani* induced 2 × TATAT to a level approximately two-thirds that of the *GRMZM2G174449* promoter in the transient system. These data indicated that GCTGA and TATAT are two *R. solani*-inducible *cis*-elements.

### Functional validation of the GCTGA and TATAT *cis*-elements in transgenic rice plants

**Fig. 7:**
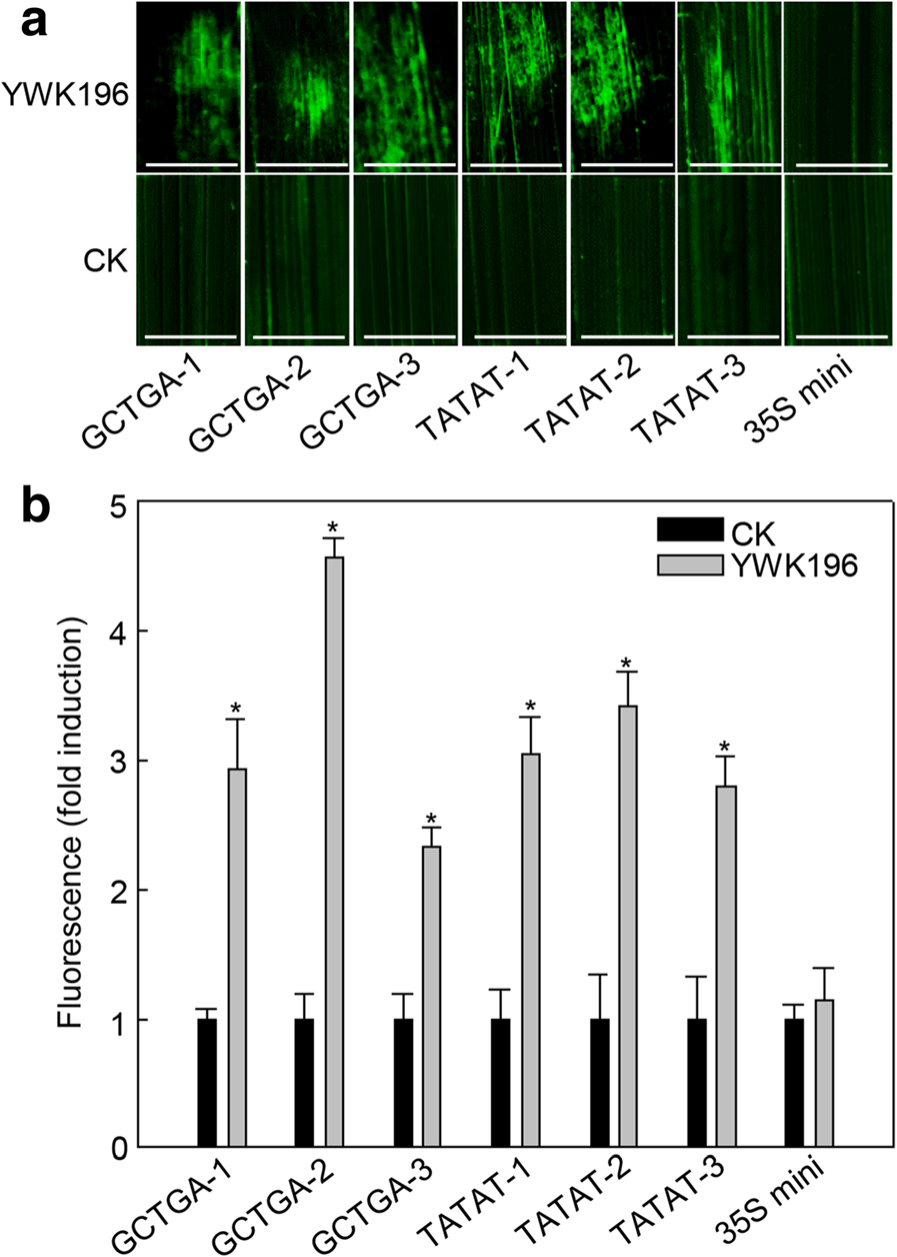
Determination of GCTGA and TATAT induction by *R. solani* in the transgenic rice leaves. **a** GFP fluorescence assay of transgenic rice leaves inoculated with *R. solani* strain YWK196. Three T_1_ lines of each element were used. Bars = 5 mm. **b** Quantitative fluorometric assay of transgenic rice leaves post inoculation with *R. solani*. The fluorescence value was calculated relative to CK of GCTGA-1. The 35S minimum promoter was used as control. Asterisks indicate statistically significant differences, as determined by Student’s t-tests (*P < 0.05)

To further confirm the results of the aforementioned transient assays, we generated GCTGA and TATAT transgenic rice plants using the constructs produced in Fig. 6. Three T_1_ lines of each element were inoculated with YWK196 for 24 h. Leaves covered with PDA medium were used as native controls. As shown in Fig. 7, strong GFP fluorescence was observed in the *R. solani*-inoculated leaves of GCTGA and TATAT transgenic rice plants, and no clear GFP fluorescence was observed in the mock leaves and native control. These results were consistent with those in the NB transient-expression assays.

To investigate whether the two *cis*-elements were responsive to other *R. solani* strains and other rice pathogens, both transgenic lines used above were inoculated with *R. solani* strains LD16 and LD21, *M. grisea* strain RB21, *Xoo* strain PXO99 and *Xoc* strain RS105 for 24 h. Leaves treated with media and water were used as controls. The GFP fluorescence was detected only in LD16- and LD21-inoculated leaves, while no considerable GFP was detected in leaves infected by other pathogens (Additional file 3: Figure S2). These results indicate that the two *cis*-elements are not responsible for the induction by *M. grisea*, *Xoo* or *Xoc.* Therefore, these two *cis*-elements might be specifically responded for *R. solani* induction.

### Determination of the key bases of the GCTGA and TATAT elements

**Fig. 8:**
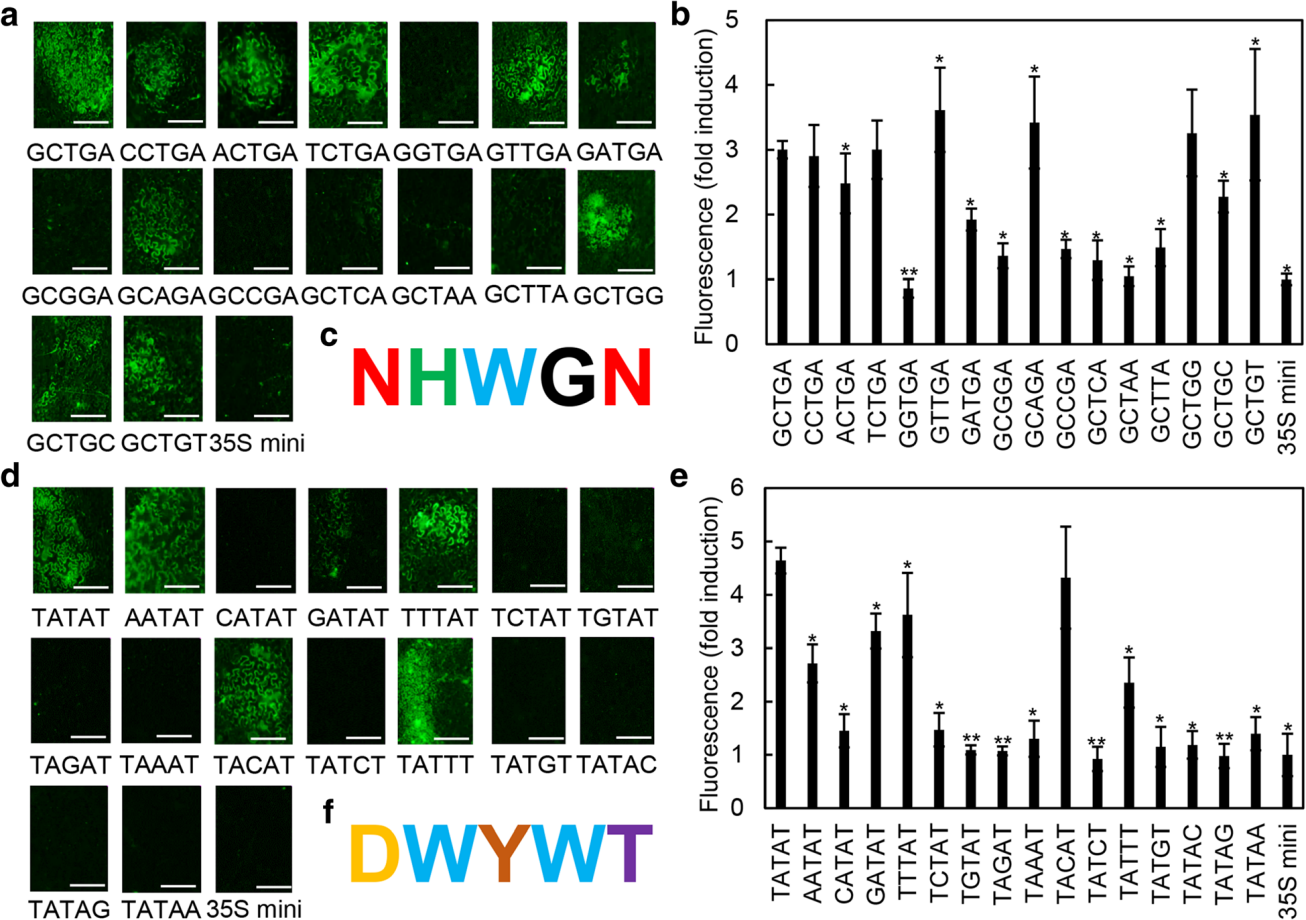
Mutation analysis of the GCTGA and TATAT elements in NB leaves. **a** GFP fluorescence assay of NB leaves expressing different GCTGA mutations after treatment with *R. solani* strain YWK196 for 24 h. Bars = 5 mm. **b** Quantitative fluorometric assay of NB leaves. The significant differences were compared with GCTGA. Asterisks indicate statistically significant differences, as determined by Student’s t-tests (*P < 0.05, **P < 0.01). **c** Conserved motif according to the GCTGA mutation analysis. N = A/T/C/G, H = A/T/C, W = A/T. **d** GFP fluorescence assay of NB leaves expressing different TATAT mutations after treatment with *R. solani* strain YWK196 for 24 h. Bars = 5 mm. **e** Quantitative fluorometric assay of NB leaves. The significant differences were compared with TATAT. Asterisks indicate statistically significant differences, as determined by Student’s t-tests (*P < 0.05, **P < 0.01). **f** Conserved motif according to the TATAT mutation analysis. D = A/T/G, W = A/T, Y = T/C

In order to identify the key bases of the two *cis*-elements, a series of point mutations were made and were fused to the 35S minimum promoter. Compared with the fusion of GCTGA element, the construct fused with the sequence of CCTGA, TCTGA or GCTGG activated the similar level of GFP fluorescence post inoculation with *R. solani*. The constructs fused with GTTGA, GCAGA or GCTGT showed enhanced GFP fluorescence (1.21-, 1.14- and 1.18-fold, respectively), while the rest mutated constructs showed significantly attenuated GFP fluorescence in transient expression assay (Fig. 8a-b). Among the GFP attenuated subgroup, the constructs fused with ACTGA, GATGA or GCTGC were most closely to the GCTGA which regard to activate the GFP expression (Fig. 8a-b). Integrating with the results above, we could summarize a conserved motif NHWGN (*N* = A/T/C/G, H = A/T/C, W = A/T) for the GCTGA *cis*-element (Fig. 8c). Compared with the construct carrying with TATAT, no mutated constructs could enhance the GFP fluorescence and only the construct of TACAT could activate the similar level of GFP fluorescence in transient expression assay. All the other mutated constructs showed significantly lessened GFP fluorescence in NB leaves (Fig. 8d-e). Among them, four mutated constructs were regarded to activate the GFP expression, including with the AATAT, the GATAT, the TTTAT and the TATTT, that is significantly higher than 35S mini and very close to the construct fused with TATAT for the GFP fluorescence quantification (Fig. 8d, e). So we could summarize a conserved motif DWYWT (D = A/T/G, W = A/T, Y = T/C) for the TATAT *cis*-element (Fig. 8f).

## Discussion

Maize is an important cereal crop and industrial raw material. With the popularization of straw returning and high-yield cultivation technique, BLSB happened seriously year by year. It has become one of the most devastated diseases on maize production. Lack of resistant resources limited the selection of BLSB resistant varieties by traditional breeding, and no major resistance genes has been identified by now. Therefore, mining *R. solani*-inducible genes and studying the regulation mechanisms of resistance could provide new insights for resistance breeding.

In this study, we isolated the promoter of a *R. solani*-inducible gene *GRMZM2G174449*. This promoter was highly induced after treatment with *R. solani* for 8 h (Fig. 2b, c), indicating that the *GRMZM2G174449* promoter is a typical *R. solani*-inducible promoter. Currently, constitutive promoters, such as the CaMV35S and ubiquitin promoters, are frequently used in the resistance improvement. However, constitutive expression of resistance genes is harmful to plant growth [2–4]. Pathogen-inducible promoters could regulate downstream genes quickly and accurately which have more benefits to breed the resistance crop than the constitutive promoters. Some *R. solani*-inducible promoters have been identified in maize and rice [30–34], and identification of the *GRMZM2G174449* promoter further supplemented the BLSB resistant resources.

Deletion analysis showed that two regions (−694 to −455 and −274 to +46) were involved in the response of *GRMZM2G174449* promoter to *R. solani* (Fig. 3a, b). According to the prediction by PLACE, a GT-1 *cis*-element and a W-box were located in the −274 to +46 region (Additional file 2: Figure S1). The GT-1 element was first identified in the *RBCS-3A* promoter [47] and previous studies showed that it could be induced by *Pseudomonas syringae* and *Phytophthora sojae* [40, 48]. Recent study also showed that the GT-1 element played important roles in the rice defense-related gene *Os2H16* responding to *R. solani* [30]. These results implied that there might be a conserved mechanism in maize and rice responding to *R. solani*, and the GT-1 signal is conserved in response to different pathogens and is one of the key factors in the basal immunity signal. The W-box is a specific binding site of WRKY transcription factors, and these transcription factors were reported to positively regulate the resistance to *R. solani* [49–51]. Overall, these results suggested that the GT-1 *cis*-element and W-box might play important roles in the −274 to +46 region.

Fine deletion analysis of the −694 to −455 region showed that the −574 to −550 and −490 to −478 were two important *R. solani*-inducible fragments (Fig. 4). In these two fragments, two elements, GCTGA and TATAT, were found to be homologous to another two identified *R. solani*-inducible *cis*-elements, GTTGA and TATTT, in the *GRMZM2G315431* promoter [34]. Further experiments showed that the GCTGA and TATAT elements exhibited *R. solani*-inducible activity and did not respond to other tested pathogens (Fig. 6, Additional file 3: Figure S2). These results suggested that GTTGA and GCTGA, TATAT and TATTT could be defined as G (T/C) TGA and TAT (T/A) T, respectively. It indicated that the two genes might be regulated expression with the conserved transcription factors that mediated a signal pathway different from the basal immunity. However, the promoter of *GRMZM2G174449* is different from the *GRMZM2G315431* promoter. The derived GFP expression showed the relative lower expression in culm and delayer induction post *R. solani* inoculation than the *GRMZM2G315431* promoter [34]. It could be partially explained that beside of two homologous *cis*-elements, there has only one additional pathogen-inducible element GT-1 in the promoter of *GRMZM2G174449* (Additional file 2: Figure S1)*,* and two GT-1 and one W-box in the promoter of *GRMZM2G315431* [34]*.* Mutation experiments showed that different mutations resulted different effects on the *R. solani*-inducible activities of the two *cis*-elements (Fig. 8a-b, d-e). According to these results, two conserved motifs NHWGN and DWYWT were summarized for the GCTGA and TATAT *cis*-elements, respectively (Fig. 8c-f). Moreover, we found that the inducible activity of GTTGA was higher than that of GCTGA, while the inducible activity of TATAT was higher than that of TATTT in the transient system (Fig. 8b-d). These results were consistent with that in the stable transgenic system (Additional file 4: Figure S3). Integrated with our recently reported two cases [30, 34], we believe that the transient system is presented good as the stable transgenic system in identifying the *R. solani*-inducible promoters. Future investigations into the interactions between the *cis*-elements and transcription factors will be meaningful, not only to improve our understanding of the molecular mechanisms of DNA-protein interactions during maize response to *R. solani*, but also for developing *R. solani*-resistant cultivars through breeding programs for maize and other crops.

## Conclusion

Banded leaf and sheath blight, a highly destructive disease caused by the soil-born pathogen *R. solani*, leads to devastating reductions in maize yield. Although efforts have been made to produce resistant maize cultivars by traditional breeding and study the interactions between maize and *R. solani*, to date, banded leaf and sheath blight is not effectively controlled in most maize planting areas and the mechanisms are still not clear. Here, we isolated the *GRMZM2G174449* promoter, and identified two DNA fragments, which could be highly induced by *R. solani*. We also mined the key *cis*-elements GCTGA and TATAT in the −574 to −550 and −490 to −478 fragments which involved in the maize response to *R. solani*. Finally, two conserved motifs NHWGN and DWYWT were summarized for the GCTGA and TATAT *cis*-elements, respectively. These findings not only enrich our knowledge of regulation of *R. solani*-inducible genes, but also provide new resources for resistance breeding.

## Additional files

Additional file 1: Table S1. Information on the PCR primers used in this study. (XLSX 12 kb)

Additional file 2: Figure S1. Schematic map of the *GRMZM2G174449* promoter with putative *cis*-elements. TSP, transcription start point; CANNTG-box, nematode-responsive box; ABA-box, ABA-responsive element; auxin-box, auxin-responsive element; GT-1-box, pathogen- and NaCl-responsive element; W-box, elicitor-responsive element; GA-box, GA-responsive element; MeJA-box, MeJA-responsive element. (TIFF 84 kb)

Additional file 3: Figure S2. GFP expression driven by GCTGA and TATAT in the transgenic rice leaves post inoculation with *R. solani* strains LD16, LD21, *M. grisea*, *Xoo* and *Xoc*. a GFP fluorescence assay of transgenic rice leaves inoculated with *R. solani* strains LD16, LD21 and *M. grisea*. Three T_1_ lines of each element were used. Bars = 5 mm. b Quantitative fluorometric assay of transgenic rice leaves post inoculation with *R. solani* strains LD16, LD21 and *M. grisea*. The 35S minimum promoter were used as the negative control. Asterisks indicate statistically significant differences, as determined by Student’s t-tests (*P < 0.05). c Three T_1_ lines of each element were inoculated with *Xoo* strain PXO99. The 35S minimum promoter was used as the negative control. d Three T_1_ lines of each element were inoculated with *Xoc* strain RS105. The 35S minimum promoter was used as the negative control. Asterisks indicate statistically significant differences, as determined by Student’s t-tests (*P < 0.05). (TIFF 2285 kb)

Additional file 4: Figure S3. Comparison of *R. solani*-inducible activities in the transgenic rice plants. a Comparison of *R. solani*-inducible activities between GCTGA and GTTGA in the transgenic rice plants. The 35S minimum promoter was used as the negative control. The significant differences were compared with GTTGA. Asterisks indicate statistically significant differences, as determined by Student’s t-tests (*P < 0.05). b Comparison of *R. solani*-inducible activities between TATAT and TATTT in the transgenic rice plants. The 35S minimum promoter was used as the negative control. The significant differences were compared with TATAT. Asterisks indicate statistically significant differences, as determined by Student’s t-tests (*P < 0.05). (TIFF 83 kb)

## Abbreviations

ABA: Abscisic acid
BHLH: Basic helix-loop-helix
BLSB: Banded leaf and sheath blight
CaMV35S: Cauliflower mosaic virus 35S
GA: Gibberellic acid
GUS: β-glucuronidase
hpi: Hours post inoculation
MeJA: Methyl jasmonate
MUG: 4-methyl-umbelliferyl-β-D-glucuronide
NB: *Nicotiana benthamiana*
qRT-PCR: Quantitative real-time PCR
RNA-seq: RNA sequencing
TSP: Transcription start point
*Xoc*: *Xanthomonas oryzae* pv. *oryzicola*
*Xoo*: *Xanthomonas oryzae* pv. *oryzae*
