# Supplementary figures and images for: Functional analysis of the GRMZM2G174449 promoter to identify Rhizoctonia solani-inducible cis-elements in maize
Source: BMC Plant Biol. 2017 Dec 4;17:233. doi: 10.1186/s12870-017-1181-5 (PMC5715495; doi:10.1186/s12870-017-1181-5)

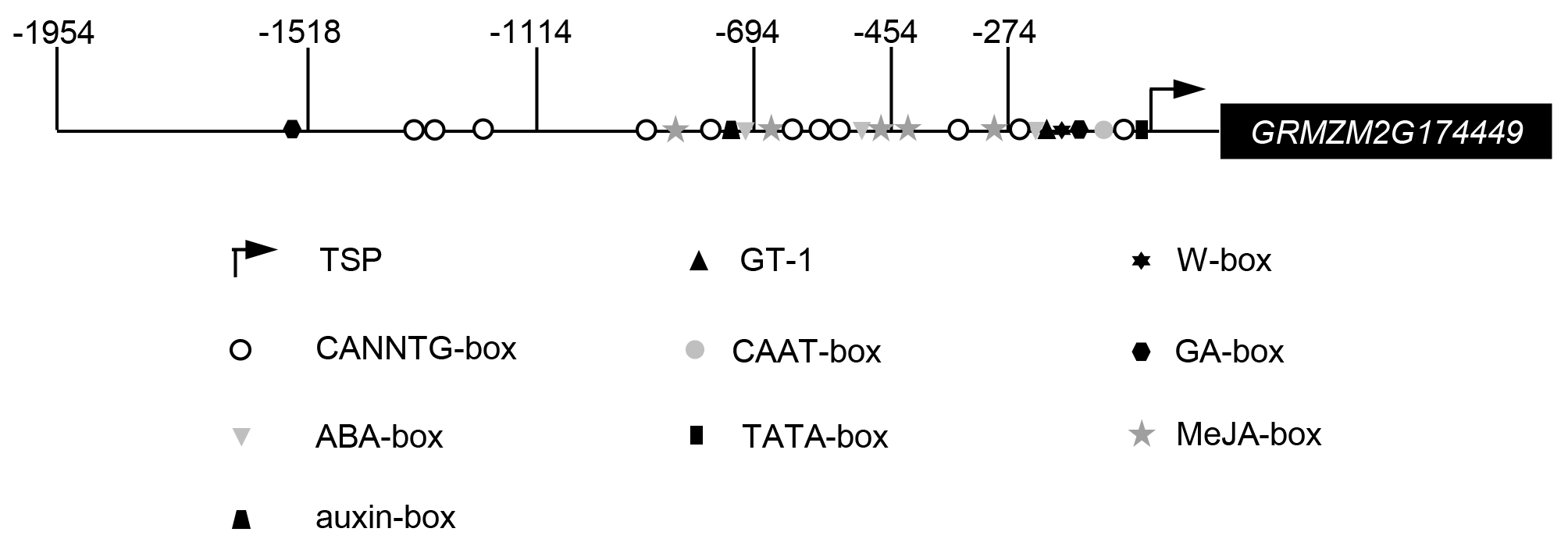

Supplement: Supplementary file 2 — Schematic map of the GRMZM2G174449 promoter with putative cis-elements. TSP, transcription start point; CANNTG-box, nematode-responsive box; ABA-box, ABA-responsive element; auxin-box, auxin-responsive element; GT-1-box, pathogen- and NaCl-responsive element; W-box, elicitor-responsive element; GA-box, GA-responsive element; MeJA-box, MeJA-responsive element. (TIFF 84 kb) [file 12870_2017_1181_MOESM2_ESM.tif]

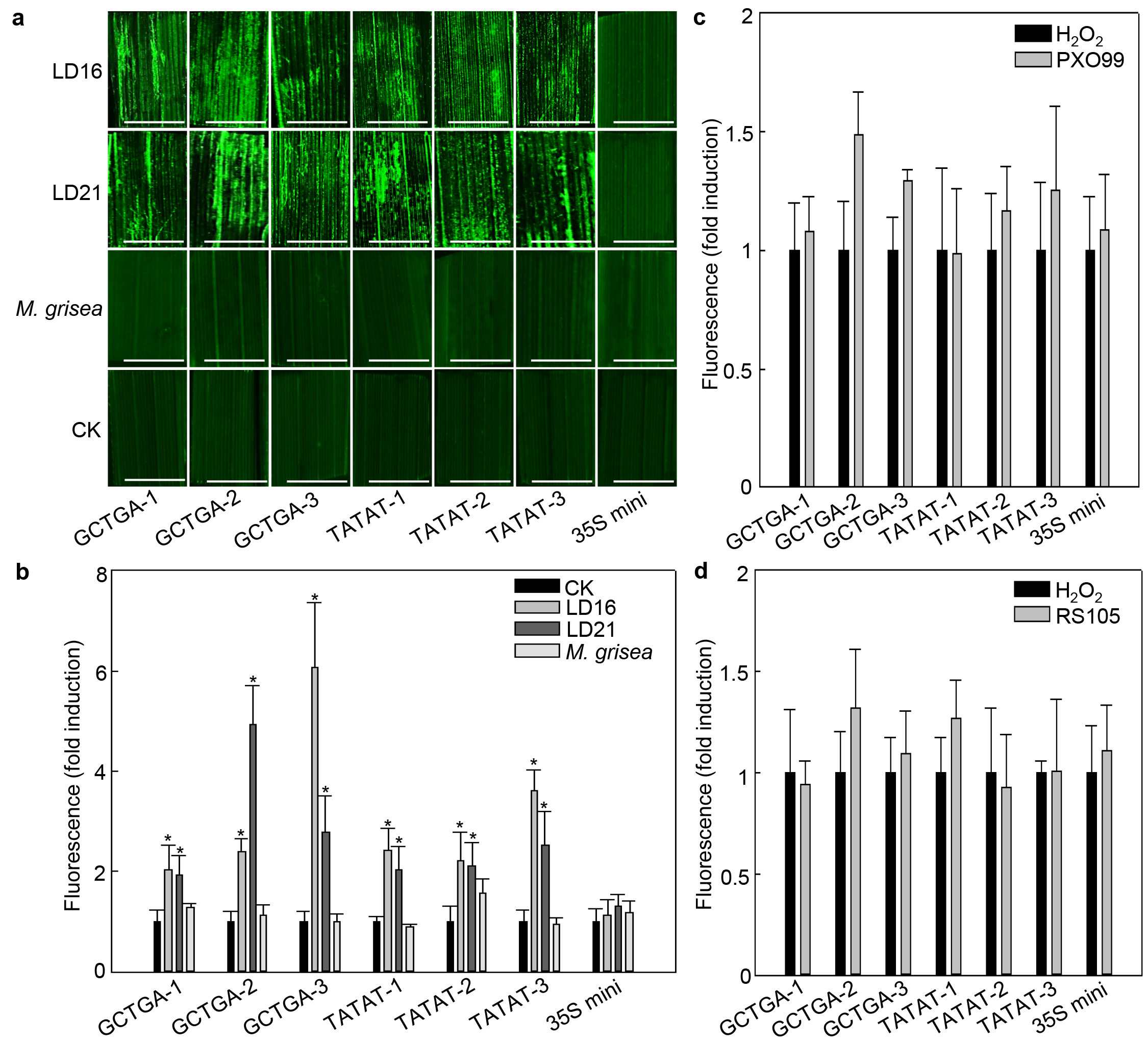

Supplement: Supplementary file 3 — GFP expression driven by GCTGA and TATAT in the transgenic rice leaves post inoculation with R. solani strains LD16, LD21, M. grisea, Xoo and Xoc. a GFP fluorescence assay of transgenic rice leaves inoculated with R. solani strains LD16, LD21 and M. grisea. Three T1 lines of each element were used. Bars = 5 mm. b Quantitative fluorometric assay of transgenic rice leaves post inoculation with R. solani strains LD16, LD21 and M. grisea. The 35S minimum promoter were used as the negative control. Asterisks indicate statistically significant differences, as determined by Student’s t-tests (*P < 0.05). c Three T1 lines of each element were inoculated with Xoo strain PXO99. The 35S minimum promoter was used as the negative control. d Three T1 lines of each element were inoculated with Xoc strain RS105. The 35S minimum promoter was used as the negative control. Asterisks indicate statistically significant differences, as determined by Student’s t-tests (*P < 0.05). (TIFF 2285 kb) [file 12870_2017_1181_MOESM3_ESM.tif]

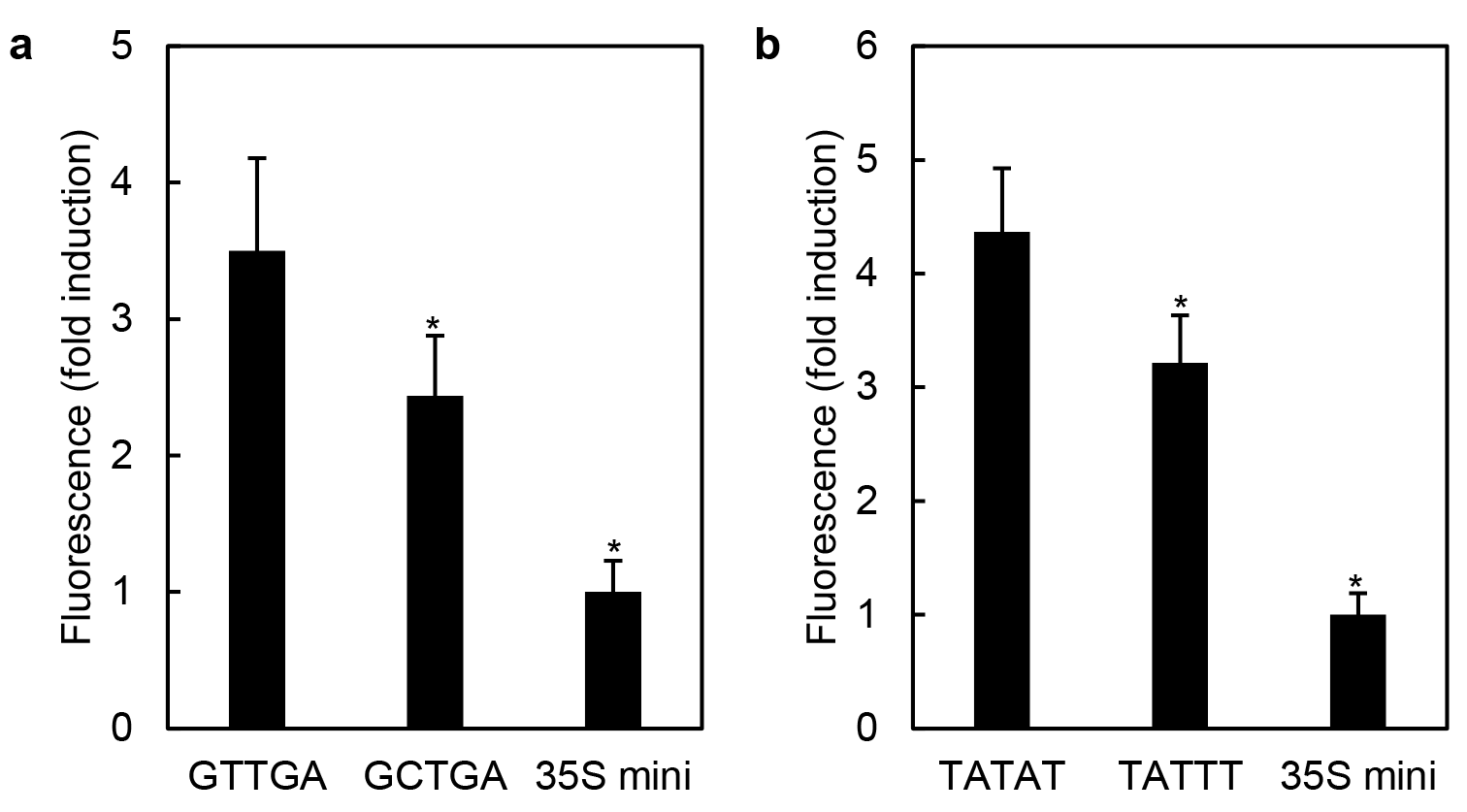

Supplement: Supplementary file 4 — Comparison of R. solani-inducible activities in the transgenic rice plants. a Comparison of R. solani-inducible activities between GCTGA and GTTGA in the transgenic rice plants. The 35S minimum promoter was used as the negative control. The significant differences were compared with GTTGA. Asterisks indicate statistically significant differences, as determined by Student’s t-tests (*P < 0.05). b Comparison of R. solani-inducible activities between TATAT and TATTT in the transgenic rice plants. The 35S minimum promoter was used as the negative control. The significant differences were compared with TATAT. Asterisks indicate statistically significant differences, as determined by Student’s t-tests (*P < 0.05). (TIFF 83 kb) [file 12870_2017_1181_MOESM4_ESM.tif]
